# Urine and serum S100A8/A9 and S100A12 associate with active lupus nephritis and may predict response to rituximab treatment

**DOI:** 10.1136/rmdopen-2020-001257

**Published:** 2020-07-28

**Authors:** Jennifer C Davies, Angela Midgley, Emil Carlsson, Sean Donohue, Ian N Bruce, Michael W Beresford, Christian M Hedrich

**Affiliations:** 1 Department of Women’s and Children’s Health, Institute of Translational Medicine, University of Liverpool, Liverpool, UK; 2 Arc Epidemiology Unit, University of Manchester, Manchester, UK; 3 Department of Rheumatology, Alder Hey Children's NHS Foundation Trust, Liverpool, UK

**Keywords:** Arthritis, Juvenile, Arthritis, Psoriatic, Cytokines, Inflammation, Lupus Erythematosus, Systemic

## Abstract

**Background:**

Approximately 30% of patients with the systemic autoimmune/inflammatory disorder systemic lupus erythematosus (SLE) develop lupus nephritis (LN) that affects treatment and prognosis. Easily accessible biomarkers do not exist to reliably predict renal disease. The Maximizing SLE Therapeutic Potential by Application of Novel and Systemic Approaches and the Engineering Consortium aims to identify indicators of treatment responses in SLE. This study tested the applicability of calcium-binding S100 proteins in serum and urine as biomarkers for disease activity and response to treatment with rituximab (RTX) in LN.

**Methods:**

S100A8/A9 and S100A12 proteins were quantified in the serum and urine of 243 patients with SLE from the British Isles Lupus Assessment Group Biologics Register (BILAG-BR) study and 48 controls matched for age using Meso Scale Discovery’s technology to determine whether they perform as biomarkers for active LN and/or may be used to predict response to treatment with RTX. Renal disease activity and response to treatment was based on BILAG-BR scores and changes in response to treatment.

**Results:**

Serum S100A12 (p<0.001), and serum and urine S100A8/A9 (p<0.001) levels are elevated in patients with SLE. While serum and urine S100 levels do not correlate with global disease activity (SLE Disease Activity Index), levels in urine and urine/serum ratios are elevated in patients with active LN. S100 proteins perform better as biomarkers for active LN involvement in patients with SLE who tested positive for anti-double-stranded DNA antibodies. Binary logistic regression and area under the curve analyses suggest the combination of serum S100A8/A9 and S100A12 can predict response to RTX treatment in LN after 6 months.

**Conclusions:**

Findings from this study show promise for clinical application of S100 proteins to predict active renal disease in SLE and response to treatment with RTX.

## INTRODUCTION

Systemic lupus erythematosus (SLE) is a systemic autoimmune/inflammatory disease that can affect any organ of the human body. The molecular pathophysiology of SLE remains largely unknown, but complex interactions of genetic factors, the environment and hormones contribute to disease expression.^1^ ^2^ Tissue inflammation and organ damage are caused by immune cell dysregulation including enhanced generation and activation of effector T cells, altered cytokine expression, B cell activation, autoantibody production, immune complex deposition and complement activation.^3^ ^4^

Approximately 30% of adult-onset patients with SLE develop renal disease,^5–7^ and damage is strongly associated with morbidity and mortality.^8^ ^9^ Diagnosing renal disease and measuring its activity can be challenging, especially since frequently tested nephritis-associated proteinuria cannot reliably predict WHO nephritis class (that centrally influences treatment decision) and/or inflammatory activity (since damage can result in renal protein loss). Thus, currently, renal biopsies are used to reliably determine renal disease and/or the form (WHO class) and extent of renal disease.^10^

Patients with SLE exhibit extraordinarily variable treatment responses, and we are currently unable to predict response to treatment options available. Though treatment recommendations for patients with SLE-associated renal disease have been published,^11^ ^12^ not all patients will respond to first-line agents and treatment optimisation not uncommonly follows trial-and-error strategies. Thus, the identification of pathomechanisms involved and the establishment of predictive biomarkers are urgently needed to optimise treatment. Indeed, biologically informed individualised and target-directed approaches promise to be of benefit to both patients and healthcare systems by reducing disease burden, time to remission, disease-associated damage and disability, and treatment-associated cost.^13^ Depletion of B lymphocytes with rituximab (RTX) can reduce SLE disease activity in a subset of patients with SLE, but only half of patients with SLE respond to RTX after 6 months.^14^ ^15^ However, biomarkers to predict treatment responses and/or disease outcomes are currently not available.

Calcium-binding S100 proteins are mainly expressed in granulocytes and mononuclear blood cells such as neutrophils and macrophages.^16^ ^17^ More recently, expression of these S100 proteins has been reported in renal endothelial cells.^18^ Elevated serum concentrations of S100 proteins have been linked with systemic inflammatory conditions in children and adults.^19^ Recently, elevated levels of pro-inflammatory S100A12 and heterodimeric S100A8/A9 in serum and urine samples from patients with juvenile-onset SLE and in serum of adult-onset patients with SLE were reported to correlate with renal disease.^20–^ ^22^ Previous studies have not examined S100 proteins in the serum and urine from the same patients and did not address the potential of combining S100 protein levels from serum and urine to determine systemic and/or renal disease status. Furthermore, the predictive value of S100 expression regarding treatment responses has not been addressed.

The MRC MASTERPLANS (major clinical response Maximizing SLE therapeutic Potential by Application of Novel and Systemic Approaches) Consortium aims to identify groups of patients who will respond to B cell depletion with RTX and identify novel predictors of treatment response. In this study, we determined serum and urine concentrations of S100 proteins S100A8/A9 and S100A12 in adult-onset SLE with or without active renal disease. Furthermore, we tested their applicability as potential markers for renal disease and disease activity as well as their capacity to predict responses to RTX treatment in patients with active lupus nephritis (LN).

## PATIENTS AND METHODS

### Patients

Serum and urine samples were collected from adult-onset patients with SLE (n=243) in the UK through centres involved in the British Isles Lupus Assessment Group (BILAG) Biologics Register (BR). The BILAG-BR received ethical approval from the Health Research Authority dated 9 November 2009 (IRAS ref. 24407) and written informed consent was obtained in accordance with the declaration of Helsinki. Serum (n=236) and urine (n=199) samples were collected at baseline in patients who were being initiated on a change in immunosuppressive/immunomodulatory treatment. Samples were sent at ambient temperature in the overnight post for processing at a central laboratory at the Centre for Musculoskeletal Research, University of Manchester. Clinical details including demographic data, concurrent treatment, disease activity and treatment response were obtained from medical records through BILAG-BR. Patients were excluded from analysis if they were taking other biologic disease-modifying anti-rheumatic drugs, and background doses of immunosuppressive drugs and corticosteroid were determined by the local treating physician according to their usual clinical practice ‘low-dose’ corticosteroids (table 1). All patients included met the 1997 American College of Rheumatology classification criteria for SLE.^23^ Furthermore, serum and urine samples were obtained from 48 adult healthy donors as controls (HC). Individual data points that required to be excluded from analysis are summarised in online supplementary table 1.

**Table 1:** Demographic and clinical information

Variable	Age-matched controls (n=48)	Patients with SLE (n=243)	P value	Patients with SLE with active renal disease (n=85)	Patients with SLE with inactive/no renal disease (n=133)	P value
Female (%)	36 (75)	222 (91)	**<0.01**	78 (92)	120 (90)	ns
Age, years (range)	38 (31–48)	40 (30.5–51)	ns	35 (25–47.5)	43 (34–53.7)	**<0.01**
Male (%)	12 (25)	21(9)	ns	7 (8)	13(10)	ns
Age, years (range)	39 (31.8–48.3)	40 (28–51)	ns	40 (29–51)	40 (30–51)	ns
Ethnicity*						
White Caucasian (%)	46 (96)	149(61)	**<0.0001**	43 (51)	93 (70)	ns
Asian (%)	2 (4)	41 (17)	-	21 (25)	17(13)	ns
Black (%)	-	29 (12)	-	11(13)	11(8)	ns
Other (%)		12 (5)		10 (11)	12 (9)	
Not specified (%)		12 (5)				
Disease duration, years (range)	-	10 (6–17)		9 (5–15)	10 (6–17)	ns
SLEDAI-2 K score (range)	-	8 (4–13)		12 (8–16)	7 (4–10)	**<0.001**
Global BILAG-BR score (range)	-	18 (12–24)		21 (14.5–28)	15 (10–22)	**<0.001**
SLICC damage score (range)	-	0 (0–1)		0 (0–1)	0 (0–2)	ns
Anti-dsDNA positivity (%)	-	122 (50)		45 (53)	54 (40)	**<0.001**
Low C3 and/or C4 (%)	-	121 (50)		49 (57)	51 (38)	**<0.01**
Antimalarial drug treatment (%)	-	230 (95)		78 (92)	120 (80)	ns
Oral corticosteroid dose, mg (range)	-	10 (9–20)		12.5 (10–20)	10 (8–20)	ns
Treatment with immunosuppressants/immunomodulators† (%)	-	102 (42)		37 (44)	50 (38)	ns
Treatment with ACE or ARB2 inhibitors (%)	-	16 (6.6)		7 (8)	8 (6)	ns

Significant differences were highlighted using bold letters.

*Ethnicity groups include white: white British, white Irish and ‘white other’; Asian: Bangladeshi, Chinese, Indian, Pakistani and ‘other Asian’; and black: black African, Caribbean and ‘other Black’.

†Immunosuppressants include azathioprine, cyclophosphamide, tacrolimus, methotrexate, mycophenolate mofetil and cyclosporin.

Anti-dsDNA, anti-double-stranded DNA; ARB2, angiotensin II receptor blockers; BILAG-BR, British Isles Lupus Assessment Group Biologics Register; ns, not significant; SLICC, Systemic Lupus International Collaborating Clinics; SLE, systemic lupus erythematosus; SLEDAI-2 K, SLE Disease Activity Index 2000.

The SLE patient cohort was separated into patients with or without active renal disease. Data are presented as total and fractions (%) for categorical variables. Median values with IQRs (25–75 IQR) are presented for continuous variables. Continuous variables were analysed using Mann-Whitney U tests; categorical variables were tested using Pearson’s χ^2^ tests.

10.1136/rmdopen-2020-001257.supp1 Supplementary data

### Classification of disease activity

The BILAG-2004 grade system scores disease activity as follows: A (severe disease), B (moderate disease), C (mild or improving disease), D (inactive disease but previous system disease) and E (system has never been involved).^24^ For data interpretation, active disease of any system was defined as having grade A or B and inactive disease as D or E. Scores of C were not included in the analysis to provide distinct disease states. To generate global BILAG-BR scores, letters A, B, C, D and E for each nine domains were converted to numerical scores, as previously described,^25^ and the sum of all domains classified as global scores. Additionally, SLE Disease Activity Index (SLEDAI)-2K^26^ scores were used. ‘No to mild’ disease activity was defined as SLEDAI scores 0–4, ‘moderate’ as scores 5–10 and ‘high to very high’ as scores ≥11.^27^ For response to RTX treatment (see the Statistical analysis section for explanation on binary regression model), lower disease activity was defined as SLEDAI scores 0–4 and lower and ≥5 as ‘higher’ disease activity, to increase numbers per group for statistical analysis.

### Definitions of treatment response

Response criteria were defined using the BILAG-BR, SLEDAI/Safety of Estrogens in Lupus Erythematosus National Assessment (SELENA SLEDAI)^28^ or SLEDAI-2000 (SLEDAI-2 K) score and steroid dose at 6- and 12-month end points. Those eligible for responder status had at least one BILAG (or BILAG-2004) A and/or 2 or more BILAG (or BILAG-2004) B scores at baseline. Response was defined by the MASTERPLANS Consortium based on criteria published previously.^14^ Patients were defined as having a ‘MCR’ if they experienced reduction in BILAG (or BILAG-2004) score to BILAG C scores in ALL domains and a reduction in steroid dose to less than or equal to 7.5 mg daily and a SLEDAI/SELENA SLEDAI or SLEDAI-2 K score less than or equal to 4 points. Patients were defined as ‘showing improvement’ (SI) if they experienced reduction in BILAG (or BILAG-2004) score to no more than one BILAG B score and no new BILAG organ domains involved and no increase in steroid dose from baseline and no increase in total SLEDAI/SELENA SLEDAI or SLEDAI-2 K score.

### Detection and quantification of S100 proteins

S100A12 homodimers and S100A8/A9 heterodimers were quantified in the serum and urine of patients with SLE and HC using Meso Scale Discovery’s Electrochemiluminescence R-plex assays (MSD, USA). Assays were performed following manufacturer’s instruction using MSD GOLD 96-well small spot streptavidin plates and recommended diluents. Final dilution factors for serum was 1:200 for S100A8/A9 and 1:100 for S100A12. Urine was diluted 1:50 and for HC 1:25 for both S100 proteins. For S100A8/A9, samples were diluted in diluent 101; for S100A12, samples were diluted in diluent 100 with a final dilution 1:2 dilution in diluent 12. All serum samples were vortexed thoroughly before use and urines were centrifuged to remove cell debris. Plates were analysed on a QuickPlex SQ 120 instrument. Urine S100 protein levels were normalised to creatinine (Cr) and presented as ng/mmolCr. For this, Cr levels were determined by the routine clinical chemistry laboratory at Alder Hey Children’s NHS Foundation Trust Hospital, using Abbott Enzymatic Creatinine assay on the Abbott Architect Ci8200 (Abbott, USA).

### Statistical analysis

Statistical analyses were performed in the software R version 3.6.0^29^ or SPSS (SPSS; IBM Corp., Armonk, NY) version 24. Graphical illustrations were generated using GraphPad Prism version 6 (Graphpad Software, San Diego, CA) or generated using R (R version 5.3.3 ‘Great Truth’, The R Foundation). All p values were considered significant at p<0.05. Because S100 protein values did not follow normal distribution, non-parametric tests were used to test statistical significance. For cross-sectional analysis between two groups, Mann-Whitney U test was used and between more than two groups, Kruskal-Wallis test with Dunn’s multiple comparison post hoc test were applied.

Binary logistic regression was conducted to examine whether S100 protein levels (all log transformed) can predict renal disease (outcome: active=1; inactive=0) and/or response toTRTX treatment (outcome: responder=1; non-responder=0). For the assessment of predictive values of S100 proteins in the presence of active renal disease (table 2), ORs, CIs and p values were first obtained for the prediction of renal disease based on S100 proteins alone for all patients. Then, patients were separated into those fulfilling any of the following: (1) anti-double-stranded DNA (anti-dsDNA) negative, (2) anti-dsDNA positive, (3) having normal serum complement and (4) having low complement and OR, CI and p values recalculated for these subgroups. To test associations of S100 protein levels with response to RTX treatment, crude and adjusted (for disease duration, disease activity, renal disease and steroid dose) ORs were calculated. For renal disease, forwards stepwise approach was used with proteins added in order of statistical significance. The ‘stepAIC’ function in R was used to determine the relative quality of models against each other. This function compares models based on all possible combinations of biomarkers and chooses the model with the minimum *Akaike* information criterion (AIC) value. The AIC is a measure of the relative quality of a model relative to each of the other models, with a lower value meaning better quality. Area under the curve (AUC) receiver operating curve (ROC) analysis was calculated for individual proteins and models, using their predicted probabilities, with outcomes ‘active renal disease’ or ‘responder to RTX’.

**Table 2:** Urine and serum S100A8/A9 and S100A12 predict renal disease in SLE

Individual models															
All patients				Anti-dsDNA negative			Anti-dsDNA positive			Normal serum complement C3 and/or C4			Low serum complement C3 and/or C4		
	OR (CI)	P value	AUC	OR (CI)	P value	AUC	OR (CI)	P value	AUC	OR (CI)	P value	AUC	OR (CI)	P value	AUC
Serum S100A8/A9	0.9 (0.62–1.3)	0.9	0.468	0.9 (0.5–1.7)	0.8	0.491	1 (0.6–1.7)	0.8	0.490	0.6 (0.3–1.2)	0.1	0.436	1.3 (0.8–2)	0.3	0.55
Serum S100A12	0.7 (0.54–0.93)	**0.015**	0.376	0.68 (0.5–0.9)	**0.042**	0.347	0.8 (0.5–1.3)	0.4	0.444	0.6 (0.4–0.9)	**0.014**	0.319	0.9 (0.6–1.4)	0.7	0.482
Urine S100A8/A9	1.36 (1.1–1.68)	**0.004**	0.623	1.2 (0.9–1.7)	0.2	0.538	1.4 (1–1.9)	**0.012**	0.687	1.3 (0.9–1.7)	0.08	0.591	1.4 (1–2)	**0.03**	0.656
Urine S100A12	1.12 (0.98–1.26)	0.078	0.590	0.9 (0.9–0.7)	0.3	0.433	1.3 (1–1.6)	**0.011**	0.685	0.9 (0.8–1.2)	0.9	0.522	1.2 (0.9–1.4)	0.07	0.63
Three-analyte model															
Urine S100A8/A9 serum S100A12 Serum S100A8/A9	1.35 (1–1.7) 0.6 (0.4–1) 1.6 (0.8–3)	**0.009** 0.052 0.16	0.681	-	-	-	-	-	-	-	-	-	-	-	-
Four-analyte model															
Urine S100A8/A9 Serum S100A12 Urine S100A12 Serum S100A8/A9	1.3 (1–1.7) 0.6 (0.3–0.99) 1.03 (0.9–1.2) 1.6 (0.8–3)	**0.048** **0.049** 0.65 0.16	0.678	1.4 (0.9–2) 0.6 (0.5–1) 0.8 (0.7–1) 1.6 (6–4)	0.09 0.09 0.2 0.3	0.708	1.3 (0.9–2) 0.9 (0.4–2.6) 1.2 (0.9–1.5) 1.1 (0.4–0.5)	0.2 0.9 0.2 0.8	0.702	1.6 (1–2.3) 0.6 (0.4–1) 0.8 (0.7–1.1) 0.9 (0.4–2.3)	**0.029** 0.07 0.2 0.9	0.715	1.3 (0.8–1.9) 0.6 (0.2–1.8) 1 (0.9–1.4) 2.3 (0.7–7.5)	0.3 0.4 0.4 0.2	0.696

Significant differences were highlighted using bold letters.

Displayed are ORs and area under the curve (AUC) analyses for S100 proteins alone and in combination with outcome of renal disease. ORs, CIs and p values are displayed.

Anti-dsDNA, anti-double-stranded DNA.

## RESULTS

### Clinical and demographic data

The study cohort included a total of 243 adult-onset patients with SLE (235 serum and 198 urine samples; 192 matched samples) and 48 HC matched for age (HC; urine and serum n=48; 46 matched samples). Clinical and demographic data are summarised in table 1. In accordance with the expectable sex distribution in the adult age group, 91% of patients with SLE included were women; median age of the cohort was (IQR) 40 (30.5–51) years, disease duration 10 (6–17) years. The median baseline total SLEDAI-2K score was 8 (4–13), global BILAG-BR score 18 (12–24) and Systemic Lupus International Collaborating Clinics damage index 0 (0–1). In the cohort, 149 (61%) were white Caucasian, 41 (17%) Indo-Asian and 29 (12%) African ancestry. Pathologically reduced serum complement C3 and/or C4 were detected in 121 (50%), and 122 (50%) were anti-dsDNA antibody positive. Systemic corticosteroids were only allowed at ‘low’ doses (<20 mg/day) and individuals taking ‘high-dose’ (>20 mg/day) corticosteroids were excluded (median dose 10 (9–20)mg). Ninety-five per cent of patients were on antimalarial treatment and approximately 7% of patients included were taking ACE inhibitors or angiotensin receptor II blockers (ARB2). Patients were divided into two groups: (1) individuals with active renal disease (n=85, renal BILAG score A 63.5%; B 36.5%) and (2) patients with inactive or no renal disease (n=133, renal BILAG score D 11%; E 89%). The age-matched HC cohort of 48 individuals included a lower proportion of women (n=36, 75%, p<0.01) when compared with the SLE cohort (n=222, 91%) and increased relative numbers of white Caucasians (n=46, 96%, p<0.0001). Individuals with active renal disease were slightly younger (35 (25–47.5), p<0.01), had higher SLEDAI-2 K (12 (8–16), p<0.001) and global BILAG-BR scores (21 (14.5–28), p<0.001) when compared with patients with inactive or no renal involvement (age; 43 (34–53.7), SLEDAI; 7 (4–10), global BILAG-BR, 15 (10–22)). Within the subcohort of individuals with active renal disease, significantly more patients were anti-dsDNA antibody positive (p<0.001) and/or had pathologically low serum complement C3 and/or C4 levels (p<0.01) when compared with patients with inactive or no renal disease. In agreement with previous reports,^30^ more patients with higher disease activity were also anti-dsDNA antibody positive and/or exhibited low complement C3 and/or C4 levels (both p<0.0001). Female patients with SLE exhibited a longer duration of disease (years; 11^6–17^) at the time of sample collection when compared with male patients with SLE (years; 6.5^3–^ ^11^; online supplementaey table 2). Demographics of subcohorts were otherwise comparable including racial distribution (online supplementary table 3). However, individuals with no or mild disease activity were significantly older than those with higher disease activity (p<0.005) (online supplementary table 4).

**Table 3:** S100 proteins predict response to RTX treatment

Shows improvement at 6 months									
	Crude OR CI		P value	Adjusted OR CI		P value	Disease activity OR CI		P value
**Serum S100A8/A9**	0.8	0.5–1.3	0.4	0.7	0.4–1.3	0.3	0.3	0.07–0.9	**0.038**
**Serum S100A12**	1	0.8–1.4	0.8	1	0.7–1.4	0.9	0.3	0.07–0.9	**0.036**
Urine S100A8/A9	1	0.8–1.4	0.6	0.9	0.7–1.4	0.9			
Urine S100A12	1	0.9–1.3	0.3	1	0.8–1.2	0.8			
Shows improvement at 12 months									
Serum S100A8/A9	0.7	0.4–1.2	0.2	0.8	0.4–1.7	0.5			
Serum S100A12	1	0.7–1.4	0.9	1	0.7–1.5	0.9			
Urine S100A8/A9	1	0.7–1.3	0.8	1	0.7–1.4	0.8			
Urine S100A12	1	0.8–1.2	0.8	0.9	0.7–1.3	0.8			
Major clinical response at 6 months									
**Serum S100A8/A9**	0.8	0.4–1.5	0.5	0.7	0.3–1.9	0.6	0.2	0.2–0.9	**0.036**
**Serum S100A12**	1	0.7–1.6	0.8	0.85	0.5–1.5	0.6	0.1	0.02–0.8	**0.03**
Urine S100A8/A9	0.99	0.7–1.4	0.9	1.1	0.7–2	0.6			
Urine S100A12	1	0.8–1.3	0.8	1.4	0.9–2	0.1			
Major clinical response at 12 months									
Serum S100A8/A9	0.8	0.4–1.5	0.5	0.8	0.4–1.9	0.6			
Serum S100A12	0.9	0.6–1.3	0.7	0.9	0.6–1.4	0.6			
Urine S100A8/A9	0.99	0.7–1.4	1	0.9	0.6–1.4	0.7			
Urine S100A12	1	0.8–1.2	0.9	0.9	0.7–1.3	0.8			

Displayed are crude and adjusted ORs for S100 proteins alone and in combination with the outcome of being a responder to RTX. ORs, CIs and p values are displayed. The ‘disease activity’ column displays OR, CI and p value for this variable when S100 proteins and other variables are included as part of the model.

Adjusted for age, disease duration, renal disease (outcome ARI), disease activity (outcome higher disease), low C3 or C4 (outcome positive), anti-dsDNA (outcome positive) and steroid dose. Bold=significant for S100 protein.

Anti-dsDNA, anti-double-stranded DNA; ARI, acute renal infarction; RTX, rituximab.

10.1136/rmdopen-2020-001257.supp2 Supplementary data

10.1136/rmdopen-2020-001257.supp3 Supplementary data

10.1136/rmdopen-2020-001257.supp4 Supplementary data

### Serum and urine S100 protein levels are elevated in patients with SLE

**Figure 1:**
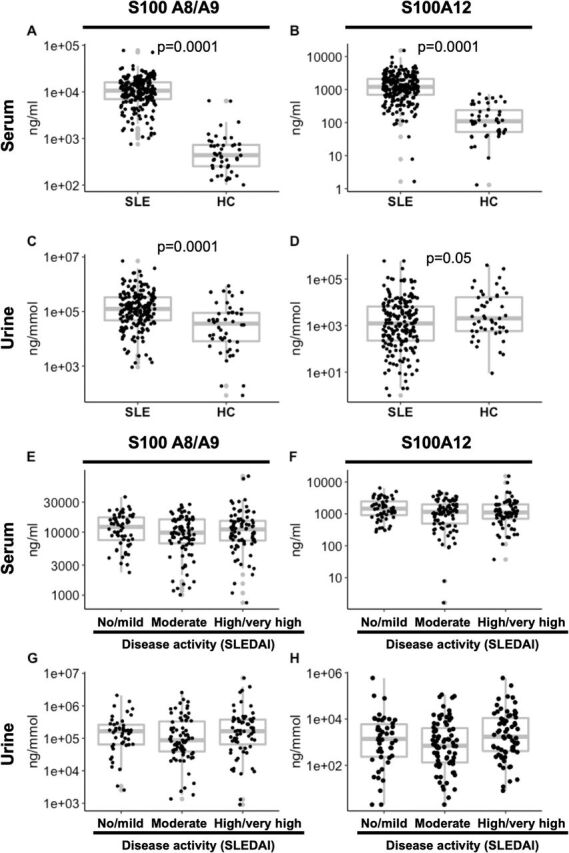
Serum and urine S100 protein levels in patients with SLE and controls. A total of 235 patients with SLE and 48 age-matched controls were analysed for S100 protein concentrations in the serum (A, B; n=235) and urine (C, D; n=192). Differences were tested using Mann-Whitney tests. Next, patients with SLE were grouped based in disease activity (E–H; SLEDAI scores: 0–4 ‘no/mild’ activity (n=61), 5–10 ‘moderate’ (n=87), and ≥11 ‘high/very high’ (n=81). S100 protein concentrations in serum (E–F; n=229) and urine (G, H; n=187) samples are displayed. Differences were tested using Kruskal-Wallis test with Dunn’s multiple comparison post hoc test were applied. HC, healthy controls; SLE, systemic lupus erythematosus; SLEDAI, SLE Disease Activity Index.

Serum S100A8/A9 and S100A12 and urine S100A8/A9 levels were significantly elevated in patients with SLE when compared with HC (p<0.0001) (figure 1A–D). Of note, urine S100A12 levels were slightly higher in HC when compared with patients with SLE (p<0.05). Serum and protein levels of S100 proteins did discriminate between healthy individuals and patients with SLE but did not associate with disease activity as measured by SLEDAI scores and was comparable across patients with no/mild, moderate and high/very high disease activity (online supplementary figure 1E–H). Furthermore, no differences in serum or urine S100 concentrations were seen between ethnicity groups included in this study (Black vs Asian vs white Caucasian vs ‘other’, not shown).

10.1136/rmdopen-2020-001257.supp6 Supplementary data

10.1136/rmdopen-2020-001257.supp7 Supplementary data

Since the presence of anti-dsDNA antibodies defines patients with ‘prototypical/classic’ SLE and serum complement C3 and/or C4 are a measure of disease activity,^30^ we tested the performance of S100 serum and protein levels in patient subgroups positive or negative for anti-dsDNA antibodies and in patients with SLE with normal versus pathologically low serum complement levels (online supplementary figure 2). Serum S100A8/A9 levels were slightly lower in patients with SLE with low serum complement C3 and/or C4 (p<0.05) when compared with individuals having normal serum complement levels. Serum S100A12 levels were also slightly lower in patients with SLE who tested positive for anti-dsDNA antibodies (p<0.05) and in patients with low serum complement C3 and/or C4 (p<0.01) when compared with individuals without these laboratory anomalies. Urine S100A8/A9 levels were not different between individuals with anti-dsDNA positivity or low serum complement C3 and/or C4. Urine S100A12 levels were significantly higher in patients with SLE with anti-dsDNA antibody positivity (p<0.05) and in patients with low serum complement C3 and/or C4 (p<0.005) as compared with those without these laboratory findings.

**Figure 2:**
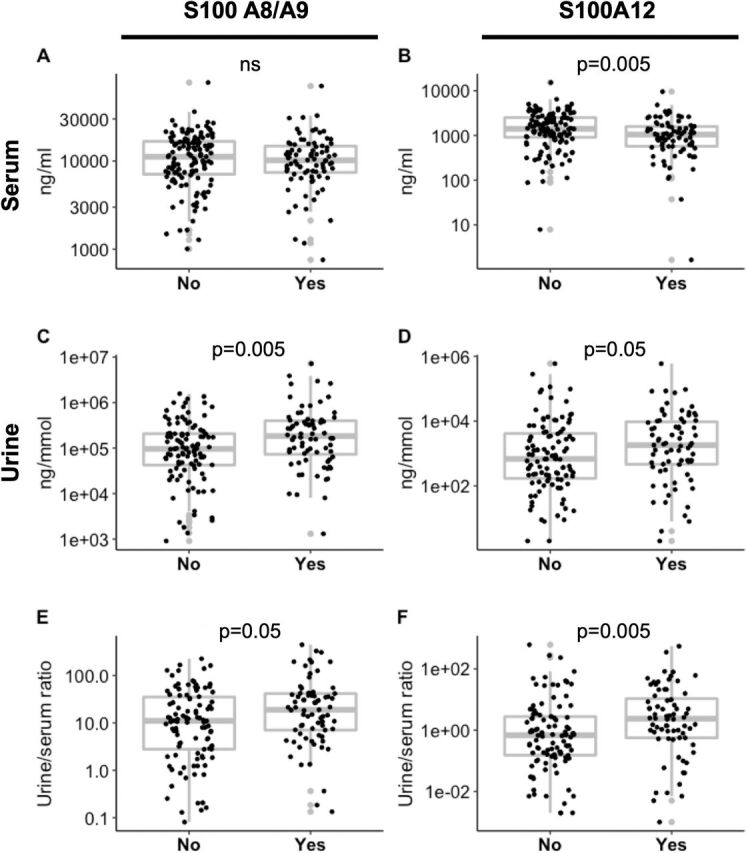
Serum and urine S100 protein levels indicate active renal disease. Serum and urine samples from 133 patients with SLE with active renal disease and from 85 patients without or inactive renal disease were included. While S100A8/A9 serum levels did not differ between (A), Serum S100A12 (B), urine S100A8/A9 (C) and S100A12 (D) levels were increased in patients with SLE with active when compared with inactive or no renal disease. Findings were reflected by S100 ratios between urine and serum (E, F). Differences were tested using Mann-Whitney U tests. SLE, systemic lupus erythematosus.

10.1136/rmdopen-2020-001257.supp8 Supplementary data

S100 protein ratios between urine and serum (urine/serum) were calculated to determine whether S100 urine levels may reflect disease activity and/or correlate with laboratory features such as low serum complement and anti-dsDNA antibody positivity. Individuals positive for anti-dsDNA antibodies and/or individuals with low serum complement C3 and/or C4 exhibited increased urine/serum ratios of S100A12. Higher urine/serum S100A8/A9 ratios were seen in individuals with low serum complement C3 and/or C4 (online supplementary figure 2E, F).

### Elevated serum and urine S100 proteins are associated with active LN

While serum S100A8/A9 levels were not different between patients with active renal disease compared with patients with SLE without or inactive renal disease (figure 2A), serum S100A12 protein levels were lower (p<0.005) in individuals with active renal disease (figure 2B). Urine S100A8/A9 and S100A12 levels were significantly higher (p<0.005 and p<0.05) in patients with active renal disease as compared with individuals with no or inactive renal disease (figure 2C, D). Furthermore, patients with SLE with active renal disease had significantly higher urine/serum ratios of S100A8/A9 (p<0.05) and S100A12 as compared with individuals with no or inactive renal disease (p<0.005) (figure 2E, F).

Patients were stratified based on the presence or absence of anti-dsDNA antibodies and/or low levels of serum complement C3 and/or C4. Using these criteria, we retested for active renal disease. Patients who tested negative for anti-dsDNA antibodies and had active renal disease exhibited lower serum S100A12 levels when compared with those with no or inactive renal disease (p<0.05) (supplement figure 3A–D). Patients tested positive for anti-dsDNA antibodies with active renal disease showed significantly higher levels of urine S100A8/A9 and S100A12 proteins as compared with those with no or inactive renal disease (both p<0.005), which was not seen in patients who tested negative for anti-dsDNA antibodies (online supplementary figure 3E, F). This may indicate renal loss of serum S100 proteins in this subgroup of patients.

10.1136/rmdopen-2020-001257.supp9 Supplementary data

Analyses after patient stratification based on low versus normal serum complement C3 and/or C4 delivered similar, however, less pronounced patterns when compared to stratification based on the presence or absence of anti-dsDNA antibodies. Patients with normal serum complement and with active renal disease had lower serum S100A12 levels when compared with those with no or inactive renal disease (p<0.01) (online supplementary figure 4B). Urine S100 proteins A8/A9 and A12 were significantly higher (p<0.05) in patients with SLE with low serum complement C3 and/or C4 and with active renal disease as compared with those without active renal involvement (online supplementary figure 4G, H).

10.1136/rmdopen-2020-001257.supp10 Supplementary data

### Urine and serum S100A8/A9 and S100A12 predict renal disease in SLE

Binary logistic regression and AUC ROC analyses were calculated to determine the potential of S100 proteins A8/A9 and A12 alone or in combination as biomarkers for renal disease in patients with SLE (table 2). Differences between groups in serum S100A12 (OR 0.7, 95% CI 0.54 to 0.93, p=0.015) and urine S100A8/A9 (OR 1.36, 95% CI 1.1 to 1.68, p=0.004) protein levels were statistically significant. The statistical model found the lowest AIC included urine S100A8/A9, serum S100A12 and serum S100A8/A9. The three-analyte model had a modest AUC of 0.681; however, urine S100A8/A9 was the only analyte significant (OR 1.35, 95% CI 1 to 1.7, p=0.009). Including all four analytes in the model delivered a slightly lower AUC of 0.678.

Stratified analyses were performed based on anti-dsDNA positivity and/or low serum complement C3 and/or C4 status. Differing analytes and combinations were seen from stratified analysis with the lowest AIC seen for serum S100A12 in patients without anti-dsDNA antibodies, urine S100A8/A9 in patients with anti-dsDNA positivity, serum and urine S100A8/A9 in patients with low serum complement C3 and/or C4 (AUC 0.672), and serum S100A12 and urine S100A8/A9 in patients with normal serum complement levels (AUC 0.705). Inclusion of all analytes in the model produced an improved AUC of 0.715 in patients with normal serum complement C3 and/or C4. Patients with low serum complement delivered an AUC of 0.696. The AUC was slightly higher in patients negative for anti-dsDNA antibodies (0.708) when compared with the whole cohort, while analysis for patients positive for anti-dsDNA exhibited the highest AUC (0.702) (table 2).

As an individual’s ethnicity can affect disease phenotype and outcome, and as white Caucasians represent the largest group of patients in this study, we tested the predictive value of serum and urine S100 protein levels to predict active LN in the white Caucasian subcohort. Indeed, results were comparable when compared with those in the entire cohort of mixed ethnicity (online supplementary table 5).

10.1136/rmdopen-2020-001257.supp5 Supplementary data

### S100A8/A9 and S100A12 proteins can predict response to treatment with RTX in a combined model

Lastly, to test whether S100 urine and/or protein levels at baseline can predict clinical response to treatment escalation with RTX in patients with active LN, S100 proteins were included in binary logistic models either alone (crude) or adjusted for confounders such as age, disease duration, renal disease (outcome ‘active renal disease’), disease activity (outcome ‘higher disease’), low serum complement C3 and/or C4 (outcome ‘positive’), the presence or absence of anti-dsDNA antibodies (outcome ‘positive’) and corticosteroid dose. Response to RTX treatment was tested in patients at 6 (n=121) and 12 months (n=85). As mentioned earlier, possible outcomes were ‘no improvement’, ‘SI’ (SI at 6 months, n=57 and 12 months, n=42) and ‘MCR’ (MCR at 6 months, n=17 and 12 months, n=21).^14^ Individual serum or urine S100 proteins at baseline did not distinguish between responders and non-responders to treatment with RTX at any time point (table 3 and online supplementary figure 5). Differences in disease activity (no response vs SI or MCR) in response to RTX were significant using a combination serum S100A8/A9 (OR 0.3, 95% CI 0.07 to 0.9) and serum S100A12 (OR 0.3, 95% CI 0.7 to 0.9) adjusted models at 6 months: Some improvement (OR 0.2, 95% CI 0.2 to 0.9), MCR (OR 0.1, 95% CI 0.02 to 0.8) (all p<0.05).

10.1136/rmdopen-2020-001257.supp11 Supplementary data

## DISCUSSION

This aim of this study, as part of the MRC MASTERPLANS Consortium, was to determine the serum and urine concentrations of S100 proteins S100A8/A9 and S100A12 in SLE to test associations with active renal disease (LN) and investigate their applicability as predictors of treatment response to B cell depletion with RTX.

Results are consistent with previous preliminary studies^12–^ ^14^ showing elevated serum S100A8/A9 and S100A12 protein levels in patients with SLE as compared with HC. No significant association was seen between global disease activity as assessed by SLEDAI scores and S100 levels in the serum or urine. Why this is remains uncertain but may be due to local production of S100 proteins in inflamed tissues rather than peripheral immune cells in the bloodstream. Indeed, S100 proteins act in an autocrine and paracrine manner amplifying inflammatory signals in the tissue microenvironment.^31–^ ^34^ Since S100 proteins are primarily expressed by innate immune cells, namely neutrophils and monocytes/macrophages, that recruit to inflamed tissues including the kidneys in SLE,^16–^ ^18^ local production may only partially translate to different serum protein levels between patients with milder versus more severe inflammatory activity in SLE.

While confirming previous reports,^12–^ ^14^ this study expands the current knowledge base significantly by providing insights into S100 urine levels and associations with disease activity and renal involvement. We found urine S100A8/A9 levels to be higher in patients with SLE as compared with age-matched HC, while urine S100A12 levels were slightly lower in patients with SLE. Associations between serum and/or urine S100 levels and organ inflammation (using the BILAG scores) only discovered activity in the renal domain (LN) to be associated with elevated S100 protein expression. This aligns with the aforementioned lack of correlation noted between S100A8/A9 and S100A12 serum or urine levels with (global) SLEDAI scores. It therefore indicates that S100 proteins may be better suited to assess renal (LN) than systemic disease activity in SLE.

Since we were interested in the applicability of S100 proteins to predict and measure renal disease activity, we stratified samples based on active renal involvement at collection. Indeed, both S100A8/A9 and S100A12 urine levels were significantly elevated in the urine of patients with SLE with active LN as compared with samples from patients with inactive or no renal disease, and calculated urine/serum ratios of both S100A8/A9 and S100A12 (from the same patients) were significantly elevated in patients with SLE with active LN. However, serum S100A12 was lower in patients with active LN, and a similar trend was previously observed (using FlexMap3D technology) in plasma for patients with SLE with a history of LN.^35^ Reduced urine S100A12 levels in the cohort including all patients with SLE (with or without active LN) and the observation that patients with SLE having active LN have lower serum S100A12 levels when compared with patients with SLE having no/inactive LN may be attributed to renal loss of S100A12 in patients with associated active LN which make up for a significant proportion of the study cohort (35%). Indeed, S100 proteins are of relatively low molecular weight (S100A8: ~11 kDa, S100A9: ~13 kDa; S100A8/A9 heterodimer: 23.9 kDa; S100A12: 21 kDa), which may result in partial ‘loss’ through filtration in healthy individuals that is further increased in LN.^36^ ^37^ Since proteins with a molecular weight between 14 and 45 kDa are partially filtered through the glomerulus and are therefore a physiological component of both the serum/plasma and urine proteome, increased filtration and associated ‘loss’ may therefore account for increased urine/serum ratios in patients with active LN.

The study cohort included in this project had a relatively high proportion of anti-dsDNA-negative individuals (50%). Anti-dsDNA positivity is frequently discussed to define ‘prototypical’ or ‘classical’ SLE phenotypes.^30^ Furthermore, pathologically reduced serum complement C3 and C4 levels correlate with disease activity and kidney involvement in SLE. Of note, stratification by reduced versus normal serum complement levels may add bias because of a preselection of cohorts with active disease. The MASTERPLANS cohort was large enough to stratify patients based on the presence of dsDNA autoantibodies that define ‘prototypical’ or ‘classical’ SLE phenotypes and to test S100 proteins in conjunction with serum complement levels. Indeed, urine S100A8/A9 and S100A12 performed better predicting renal involvement in anti-dsDNA-positive patients with SLE and in individuals with low serum complement C3 and/or C4 levels. While no differences were seen in serum levels of either protein, urine S100 levels were significantly elevated in anti-dsDNA-positive individuals with active LN. This indeed further suggests local renal production and loss of S100A8/A9 and S100A12 (by epithelia or tissue immune cells) in LN since serum levels were comparable between individuals with active or no LN. Of note, findings only partially correlated with previous (but significantly smaller) studies that saw differences in serum S100 protein levels for those with low levels of C3/C4 but did see increased levels of serum S100A8/A9 (trend for serum S100A12) who were anti-dsDNA positive.^20^ ^22^

The overall goal of the MASTERPLANS Consortium is to identify groups of patients who will respond to treatment with RTX, enabling patient stratification and individualised treatment. To test the predictive value of S100 serum and urine levels on treatment response, we applied binary regression models, testing individual variables (serum or urine S100A8/A9 and S100A12) as well as combinations of these. Indeed, serum S100A8/A9 and serum S100A12 adjusted models promise potential for the prediction of ‘(showing) improvement’ at 6 months, and serum S100A8/A9 and serum S100A12 adjusted models may predict ‘MCR’ at 6 months.^14^

While results from this study are exciting and promise potential for the use of S100 proteins predicting renal involvement and response to treatment (with RTX), as ranges of data points overlap between different outcome groups, it is difficult or currently not possible to reliably define cut-offs for future clinical use. Prospective studies in independent cohorts are necessary to validate findings, to test the performance of S100 proteins as measures of current disease activity and for the prediction of flares in disease. They are also needed to further investigate whether elevated S100 protein levels only associate with response to RTX treatment or also with other B cell depleting or other non-B cell-directed immune therapies in SLE. Furthermore, S100 proteins require to be tested in combination with additional biomarkers/mediators of systemic and/or organ inflammation, such as type I interferon expression, autoantibody patterns, genetic factors.

This study has some notable limitations. Though involving a large cohort of patients carefully monitored, some data points were missing and therefore needed to be excluded from analyses. Overall, the control cohort (n=48) is smaller as compared with the SLE patient cohort (n=243) and included a higher proportion of men (25% vs 9%) and white Caucasians (96% vs 61%). As an individual’s ethnicity can affect the risk for developing LN and its severity, we tested the ability of S100 proteins to predict the presence of LN in the White Caucasian subcohort of the study population. Models for predicting active LN performed similarly in this smaller, but ethnically homogenous group when compared with the larger ethnically diverse group. Furthermore, S100 serum and urine levels did not vary between races, and all subgroups of ethnicities among patients with SLE showed significantly elevated levels of serum S100A12 and S100A8/9, and urine S100A8/9 (data not shown).

As women may have elevated white and red blood cell count in urine contributing to leucocyte-derived inflammatory (such as S100) protein expression, the unequal gender ratio in the patient group may affect results when compared with the control cohort. Indeed, we saw increased levels of urine S100A8/9 in women as compared with men in both patients and HC. However, consistent with overall results, significant differences in urine S100A8/9 levels between patients and HC remained when excluding male samples from the analysis (data not shown). Renal disease activity was assessed using renal BILAG scores, and detailed contemporaneous biopsy reports were not available in all cases. However, data were collected through a selected set of medical institutions very experienced in the assessment of SLE disease activity and BILAG scoring (BILAG-BR centres). Nonetheless, kidney biopsies may offer an additional level of confidence and would furthermore allow associations between S100 protein expression and WHO class of LN. In this study, only S100A8/A9 and S100A12 proteins were included. It may be beneficial to include additional S100 family members in future studies, such as S100A4.^21^

## CONCLUSIONS

Serum S100A12, and serum and urine S100A8/A9 are elevated in patients with SLE when compared with HC. While S100 levels do not correlate with global disease activity, urine protein levels and urine/serum ratios enable us to predict renal involvement. Tests perform better in ‘prototypical/classical’ patients with SLE who tested positive for anti-dsDNA antibodies. A combination of serum S100A8/A9 and S100A12 may predict response to RTX treatment after 6 months. While findings are encouraging and promise clinical applications, significantly overlapping values between groups currently prohibit the definition of cut-off values and prospective studies are required to validate findings.

**Key messages**

**What is already known about this subject?**

- Though ≈30% of patients with Systemic lupus erythematosus (SLE) develop lupus nephritis that affects treatment and prognosis, easily accessible biomarkers do not exist to reliably predict renal disease.

**What does this study add?**

- Serum S100A12, and serum and urine S100A8/A9 (p<0.001) levels are elevated in SLE patients, and levels in urine and urine/serum ratios are elevated in patients with active LN.
- Binary logistic regression and AUC analyses suggests the combination of serum S100A8/A9 and S100A12 can predict response to RTX treatment in LN after 6 months.

**How might this impact on clinical practice?**

- While findings show promise for clinical application of S100 proteins to predict active LN and response to treatment with rituximab, they require to be confirmed in independent cohorts and longitudinal/prospective studies.

**Lay summary**

**What is the research on?**

- In this study, the levels of two proteins in the urine and serum of patients with SLE and age-matched healthy controls were measured. The aim of doing this was to develop markers of active disease and/or kidney involvement in SLE and to test whether they predict response to treatment with rituximab.
- The molecules measured (S100A8/A9 and S100A12) belong to a group of proteins (S100) that have many functions in regulating how cells grow, mature, die and move. Furthermore, they are involved in inflammation and immune responses. Their increased expression has been observed in other autoimmune/inflammatory conditions, including rheumatoid arthritis and juvenile idiopathic arthritis, where they further increase during periods of disease flares.

**What does this research teach scientists?**

- Both proteins, S100A8/A9 and S100A12, are elevated in the serum of patients with SLE when compared with age-matched healthy controls; S100A8/A9 is elevated in the urine of patients as compared with age-matched controls. Furthermore, patients with active kidney disease exhibit higher levels of S100A8/A9 in their urine when compared with patients without active kidney involvement. This appears to be specific to kidney disease as the expression of these proteins did not correlate with any other organ involvement. Of note, the elevated levels of S100 proteins more reliably associate with kidney involvement in patients that test positive for anti-double-stranded DNA (dsDNA) antibodies and/or have pathologically low serum complement levels. Furthermore, a statistical model allows the prediction of response to treatment with rituximab, a treatment targeting certain immune cells (B cells).
- Taken together, results from this study promise potential for S100 proteins as biomarkers for renal involvement in SLE. Furthermore, they may deliver new tools for stratifying patients with SLE to assess risk, predict individual outcomes and offer better and safer treatments.

**What difference will this make to lupus patients’ lives?**

- Observations from this study provide preliminary evidence for a minimally invasive test (urine sample and/or blood test vs kidney biopsy) that may allow the prediction of active kidney disease and response to treatment with rituximab. After validation of findings in larger, unrelated and international cohorts, S100 proteins may serve as biomarkers for an unbiased assessment of active renal involvement and treatment responses in SLE. This may reduce the number of kidney biopsies performed, reduce cost and allow quicker induction of remission with reduced side-effects through individualised treatment.

**Is further research needed or are your results strong enough?**

- Findings have to be confirmed in independent multi-ethnic cohorts and with alternative analytic procedures before they can be recommended as routine laboratory test in clinical settings.
- While results are very encouraging, individual serum or urine S100 proteins alone were relatively weak predictors of response to rituximab treatment. Since combinations with other markers of inflammation may improve robustness, results from this study will be included in the MASTERPLANS predictive algorithms including a host of additional laboratory and clinical markers.
