# Supplementary material for: Urine and serum S100A8/A9 and S100A12 associate with active lupus nephritis and may predict response to rituximab treatment
Source: RMD Open. 2020 Jul 28;6(2):e001257. doi: 10.1136/rmdopen-2020-001257 (PMC7722276; doi:10.1136/rmdopen-2020-001257)
Supplement: Supplementary data [file rmdopen-2020-001257supp001.pdf]

Supplement table 1. Missing clinical datasets.

**Clinical variable**	**Missing cases (% of total)**
Age	14 (5.7)
Ethnicity	1 (0.4)
Disease duration, years	5 (2)
SLEDAI-2K score	1 (5.3)
Global BILAG-BR score	29 (12)
SLICC damage score	19 (7.8)
Anti-ds-DNA positivity	13 (5.3)
Low C3 and/or C4	13 (5.3)
Renal BILAG-BR score A/B	25 (10.2)

Missing data are presented as percentage of the total SLE cohort (n=243).
