# Supplementary material for: Urine and serum S100A8/A9 and S100A12 associate with active lupus nephritis and may predict response to rituximab treatment
Source: RMD Open. 2020 Jul 28;6(2):e001257. doi: 10.1136/rmdopen-2020-001257 (PMC7722276; doi:10.1136/rmdopen-2020-001257)
Supplement: Supplementary data [file rmdopen-2020-001257supp002.pdf]

<u>Supplement table 2:</u> Demographic features comparing male and female SLE patients.

Variable	SLE male	SLE female	p
Age, years (range)	44 [44-52]	39 [30-51]	ns
Ethnicity[1]			
White Caucasian (%)	3 (17.6)	26 (13)	ns
Asian (%)	5 (29.4)	34 (17)	ns
Black (%)	9 (53)	199 (70)	ns
Disease duration, years (range)	6.5 [3-11]	11 [6-17]	***<0.01***
SLEDAI-2K score (range)	8 [4.5-11.5]	8 [4-13.25]	ns
Global BILAG-BR score (range)	20.5 [13.25-24]	18 [12-24]	ns
SLICC damage score (range)	1 [0-2]	0 [0-1]	ns
Anti-ds-DNA positivity (%)	9 (45)	99 (47)	ns
Low C3 and/or C4 (%)	13 (65)	102 (48)	ns
Antimalarial drug treatment (%)	19 (90)	201 (90)	ns
Oral corticosteroid dose, mg (range)	20 [7.75-20]	10 [10-20]	ns
Treatment with immunosuppressants /immunomodulators[2] (%)	10 (48)	92 (41)	ns
Treatment with ACE or ARB2 inhibitors (%)	1 (4.7)	15 (6.7)	ns
Renal BILAG-BR score A/B (%)	7 (35)	78 (39)	ns

Data are presented as total and fractions (%) for categorical variables. Median values with interquartile ranges [25-75 IQR] are presented for continuous variables. Continuous variables were analysed using Mann Whitney U tests; categorical variables were tested using Pearson's Chi-square tests.ns= not significant

[1] Ethnicity groups include White: White British, white Irish and "white other"; Asian: Bangladeshi, Chinese, Indian, Pakistani and "other Asian"; and Black: Black African, Caribbean and "other Black".

[2] Immunosuppressants include Azathioprine, Cyclophosphamide, Tacrolimus, Methotrexate, Mycophenolate Mofetil and Cyclosporin.

ACE- Angiotensin-converting enzyme and ARB2 - Angiotensin II receptor blockers
