# Supplementary material for: Urine and serum S100A8/A9 and S100A12 associate with active lupus nephritis and may predict response to rituximab treatment
Source: RMD Open. 2020 Jul 28;6(2):e001257. doi: 10.1136/rmdopen-2020-001257 (PMC7722276; doi:10.1136/rmdopen-2020-001257)
Supplement: Supplementary data [file rmdopen-2020-001257supp003.pdf]

Supplement table 3: **Demographic and clinical information.**

Variable	White Caucasian (n=149)	Asian (n=41)	Black (n=29)	Other (n=12)	Not specified (n=12)
Female (%)	140 (95)	36 (88)	26 (90)	9 (75)	11 (92)
Age, years (range)	40 [31-51]	40 [30-51]	40 [29-50.5]	40 [29-50.25]	40 [30-50]
Male (%)	9 (5)	5 (12)	3 (10)	3 (25)	1(8)
Age, years (range)	41[31-51]	41 [31-50]	42 [33.25-50]	41 [30-51]	44
Disease duration, years (range)	10 [6-17]	10[6-17]	10 [6-16]	10 [5-16]	10 [6-16]
SLEDAI-2K score (range)	8[4-13]	8[4-14]	8[4-12]	8[4-12]	8 [4-12]
Global BILAG-BR score (range)	18.5 [12.5-24]	18[12-24]	18[12-24]	17[12-24]	18 [12.75-24]
SLICC damage score (range)	0 [0-1]	0 [0-1]	0 [0-1]	0 [0-1]	0 [0-1]
Anti-ds-DNA positivity (%)	60 (40)	25 (61)	15 (51)	6 (50)	3 (25)
Low C3 and/or C4 (%)	67 (45)	23 (56)	14 (48)	7 (58)	5 (42)
Antimalarial drug treatment (%)	136 (91)	37 (90)	27 (93)	10 (83)	10 (83)
Oral corticosteroid dose, mg (range)	10 [10-20]	10 [9.5-20]	10 [10-20]	10 [9-20]	10 [10-20]
Treatment with immunosuppressants /immunomodulators[2]	59 (40)	23 (56)	11 (38)	5 (42)	4 (33)
Treatment with ACE or ARB2 inhibitors (%)	10 (6.7)	4(9.7)	2 (6.9)	0 (0)	0 (0)

The SLE patient cohort was grouped by ethnicity. Data are presented as total and fractions (%) for categorical variables. Median values with interquartile ranges [25-75 IQR] are presented for continuous variables. Continuous variables were analysed using Mann Whitney U tests; categorical variables were tested using Pearson's Chi-square tests. While women and White Caucasian origin were (expectedly) overrepresented, no significant differences in the variables investigated were identified between races or genders.

[1] Ethnicity groups include White: White British, white Irish and "white other"; Asian: Bangladeshi, Chinese, Indian, Pakistani and "other Asian"; and Black: Black African, Caribbean and "other Black".

[2] Immunosuppressants include Azathioprine, Cyclophosphamide, Tacrolimus, Methotrexate, Mycophenolate Mofetil and Cyclosporin.

ACE- Angiotensin-converting enzyme and ARB2 - Angiotensin II receptor blockers
