# Supplementary material for: Urine and serum S100A8/A9 and S100A12 associate with active lupus nephritis and may predict response to rituximab treatment
Source: RMD Open. 2020 Jul 28;6(2):e001257. doi: 10.1136/rmdopen-2020-001257 (PMC7722276; doi:10.1136/rmdopen-2020-001257)
Supplement: Supplementary data [file rmdopen-2020-001257supp004.pdf]

Supplement table 4: Demographic features based on SLEDAI disease activity.

**Variable**	**No/mild activity**	**Moderate activity**	**High/very high activity**	**p**
Female	56 (26)	77 (36)	77 (36)	ns
Male	5 (25)	10 (50)	5 (25)	ns
Age, years	44 [31-55]	43 [34-52]	36 [26-43]	***p<0.005*[3]**
Ethnicity[1]				
White Caucasian	4 (15)	10 (37)	13 (48)	ns
Asian	12 (30)	7 (19)	17 (47)	ns
Black	40 (28)	57 (40)	44 (31)	ns
Disease duration, years	11.5 [6-19]	8 [5-14]	10 [7-16]	ns
SLICC damage score	0 [0-1]	0 [0-1]	0 [0-2]	ns
Anti-ds-DNA positivity	8 (7.4)	41 (38)	59 (54.6)	***<0.0001*[3]** ***<0.05*[3]**
Low C3 and/or C4	8 (7)	39 (36)	62 (57)	***<0.0001*[3]** ***<0.05*[3]**
Antimalarial drug treatment	56 (27)	76 (36)	77 (37)	ns
Treatment with immunosuppressants /immunomodulators[2]	28 (30)	29 (32)	35 (38)	ns
Oral corticosteroid dose, mg	10 [9-20]	11 [10-20]	10 [5-19]	ns
Treatment with ACE or ARB2 inhibitors	3 (17)	6 (38)	7 (44)	ns

Low disease activity was defined as SLEADI scores 0-4, moderate as scores 5-10 and high to very high as 11. Data are presented as total and fractions (%) for categorical variables. Median values with interquartile ranges [25-75 IQR] are presented for continuous variables. Continuous variables were analysed using Mann Whitney U tests; categorical variables were tested using Pearson's Chi-square tests. ns= not significant

[1] Ethnicity groups include White: White British, white Irish and "white other"; Asian: Bangladeshi, Chinese, Indian, Pakistani and "other Asian"; and Black: Black African, Caribbean and "other Black".

[2] Immunosuppressants include Azathioprine, Cyclophosphamide, Tacrolimus, Methotrexate, Mycophenolate Mofetil and Cyclosporin.

[3] multiple comparisons between no-mild vs moderate p<0.05 and moderate–high vs very high p<0.005.

ACE: Angiotensin-converting enzyme; ARB2: Angiotensin II receptor blockers
