# Supplementary figures and images for: Urine and serum S100A8/A9 and S100A12 associate with active lupus nephritis and may predict response to rituximab treatment
Source: RMD Open. 2020 Jul 28;6(2):e001257. doi: 10.1136/rmdopen-2020-001257 (PMC7722276; doi:10.1136/rmdopen-2020-001257)

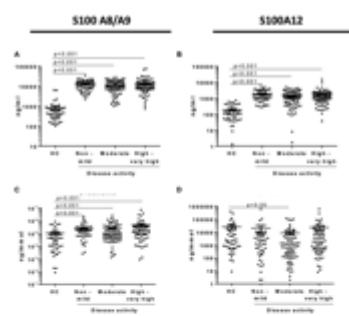

Supplement figure 1

Supplement: Supplementary data [file rmdopen-2020-001257supp007.pdf]

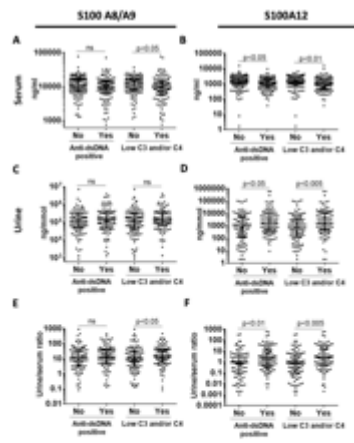

Supplement Figure 2

Supplement: Supplementary data [file rmdopen-2020-001257supp008.pdf]

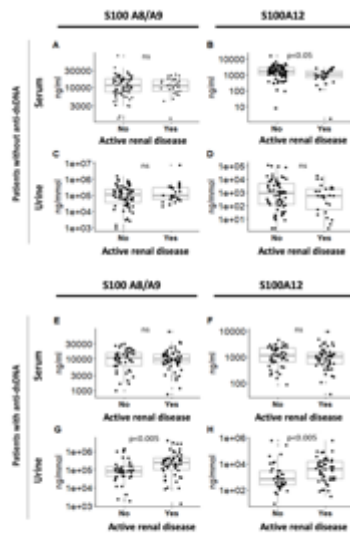

Supplement Figure 3

Supplement: Supplementary data [file rmdopen-2020-001257supp009.pdf]

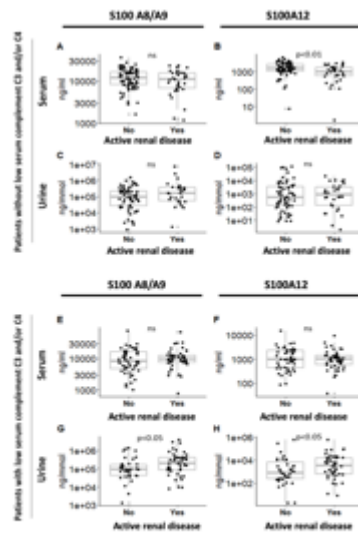

Supplement Figure 4

Supplement: Supplementary data [file rmdopen-2020-001257supp0010.pdf]

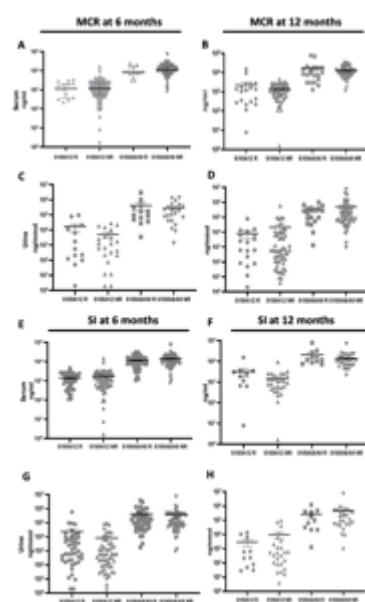

Supplement figure 5

Supplement: Supplementary data [file rmdopen-2020-001257supp0011.pdf]
