# Supplementary material for: Urine and serum S100A8/A9 and S100A12 associate with active lupus nephritis and may predict response to rituximab treatment
Source: RMD Open. 2020 Jul 28;6(2):e001257. doi: 10.1136/rmdopen-2020-001257 (PMC7722276; doi:10.1136/rmdopen-2020-001257)
Supplement: Supplementary data [file rmdopen-2020-001257supp005.pdf]

Supplement table 5: **Urine and serum S100A8/A9 and S100A12 predict renal disease in White Caucasian SLE patients.**

Individual models															
**White Caucasian patients**				**Anti-dsDNA negative**			**Anti-dsDNA positive**			**Normal serum complement C3 and/or C4**			**Low serum complement C3 and/or C4**		
	**OR (CI)**	**p value**	**AUC**	**OR (CI)**	**p value**	**AUC**	**OR (CI)**	**p value**	**AUC**	**OR (CI)**	**p**	**AUC**	**OR (CI)**	**p**	**AUC**
Serum S100A8/A9	0.79<br>(0.46-1.4)	0.414	0.451	0.85<br>(0.33-2.2)	0.727	0.457	0.76<br>(0.35-1.7)	0.494	0.439	0.85<br>(0.31-2.3)	0.743	0.464	0.99<br>(0.49-2.0)	0.981	0.500
Serum S100A12	0.60<br>(0.41-0.89)	***0.010***	0.323	0.61<br>(0.38-0.98)	***0.041***	0.265	0.61<br>(0.33-1.1)	0.119	0.374	0.59<br>(0.36-0.96)	***0.033***	0.281	0.72<br>(0.39-1.3)	0.286	0.426
Urine S100A8/A9	1.5<br>(1.1-2.1)	***0.008***	0.644	1.2<br>(0.75-1.8)	0.493	0.540	1.8<br>(1.1-2.9)	***0.020***	0.719	1.3<br>(0.86-2.0)	0.203	0.588	1.7<br>(1.0-2.9)	***0.036***	0.696
Urine S100A12	1.2<br>(1.0-1.4)	***0.028***	0.628	1.0<br>(0.80-1.3)	0.814	0.518	1.3<br>(0.98-1.7)	0.071	0.664	1.1<br>(0.86-1.4)	0.457	0.581	1.2<br>(0.96-1.6)	0.098	0.654
**Three analyte panel**															
Urine S100A8/A9<br>serum S100A12<br>Serum S100A8/A9	1.5 (1.1-2.1)<br>0.59 (0.35-1.0)<br>1.2 (0.55-2.8)	***0.018***<br>0.051<br>0.597	0.726	1.2 (0.72-2.1)<br>0.57 (0.33-0.99)<br>0.92 (0.29-2.9)	0.444<br>***0.044***<br>0.891	0.730	1.7 (1.0-2.9)<br>0.83 (0.26-2.7)<br>0.99 (0.21-4.8)	0.052<br>0.751<br>0.99	0.733	1.3 (0.79-2.0)<br>0.58 (0.34-1.0)<br>1.1 (0.28-3.9)	0.333<br>***0.049***<br>0.932	0.712	1.7 (0.97-3.0)<br>0.75 (0.20-2.8)<br>1.3 (0.30-6.1)	0.064<br>0.660<br>0.700	0.742
**Four analyte model**															
Urine S100A8/A9<br>Serum S100A12<br>Urine S100A12<br>Serum S100A8/A9	1.2 (0.84-1.9)<br>0.58 (0.35-0.97)<br>1.2 (0.94-1.5)<br>1.2 (0.52-2.7)	0.279<br>***0.039***<br>0.15<br>0.696	0.740	1.0 (0.55-2.0)<br>0.54 (0.31-0.96)<br>1.2 (0.82-1.7)<br>0.87 (0.27-2.7)	0.903<br>***0.037***<br>0.355<br>0.813	0.746	1.6 (0.82-3.0)<br>0.84 (0.26-2.7)<br>1.1 (0.75-1.5)<br>0.98 (0.20-4.7)	0.169<br>0.768<br>0.699<br>0.979	0.749	1.1 (0.62-2.1)<br>0.57 (0.33-0.99)<br>1.1 (0.76-1.6)<br>1.0 (0.27-3.8)	0.684<br>***0.044***<br>0.614<br>0.986	0.716	1.5 (0.80-2.6)<br>0.74 (0.19-2.9)<br>1.2 (0.86-1.6)<br>1.3 (0.27-6.0)	0.225<br>0.664<br>0.308<br>0.766	0.762

Displayed are odds ratios and area under the curve analyses (AUC) for S100 proteins alone and in combination with outcome of renal disease. Odds ratios (OR), confidence intervals (CI) and p values are displayed.
